# I can see my virtual body in a mirror: The role of visual perspective in changing implicit racial attitudes using virtual reality

**DOI:** 10.3389/fpsyg.2022.989582

**Published:** 2022-11-28

**Authors:** Maddalena Marini, Antonino Casile

**Affiliations:** ^1^Center for Translational Neurophysiology of Speech and Communication, Istituto Italiano di Tecnologia, Ferrara, Italy; ^2^Department of Psychology, University of Campania Luigi Vanvitelli, Caserta, Italy; ^3^Department of Mathematics and Computer Science, University of Ferrara, Ferrara, Italy

**Keywords:** implicit attitudes, race/ethnicity, virtual reality, implicit association test, malleability, visual perspective, virtual mirror

## Abstract

**Introduction:**

Recent studies showed that VR is a valid tool to change implicit attitudes toward outgroup members. Here, we extended this work by investigating conditions under which virtual reality (VR) is effective in changing implicit racial attitudes.

**Methods:**

To this end, participants were embodied in a Black or White avatar and we manipulated the perspective through which they could see their virtual body. Participants in one condition, could see their virtual body both from a first-person perspective (i.e., by looking down toward themselves) and reflected in a mirror placed in front of them in the VR environment. Participants in another condition could instead see their virtual body only from a first-person perspective (i.e., by looking down toward themselves) as no mirror was placed in the VR environment. Implicit racial attitudes were assessed using the Implicit Association Test (IAT) before and immediately after the VR intervention.

**Results:**

Results showed that when White participants were embodied in a Black avatar compared to a White avatar, they showed a decrease in their implicit pro-White attitudes but only when they could see their virtual body both from a first-person perspective and in a mirror.

**Discussion:**

These results suggest that, in immersive virtual reality interventions, the possibility for participants to see their body also reflected in a mirror, might be a critical factor in changing their implicit racial attitudes.

## Introduction

Thinking about others and making inferences about them is a spontaneous and automatic process of the human mind (Mitchell et al., 2002; Mitchell, 2009). Our brain is specialized in drawing inferences about other people – about their thoughts and feelings, their beliefs and intentions, their abilities and attributes – based on the social group to which they belong (Mitchell et al., 2006; Marini and Banaji, 2022a). We tend to display positive attitudes and act positively toward people that we deem more “similar” to us (in-group members), and display negative attitudes and act negatively toward people who are not similar to us (out-group members; Tajfel et al., 1971; Hewstone et al., 2002).

Although making inferences about others can be viewed as a useful cognitive process to quickly understand a situation and inform our behavioral responses (Allport, 1954), studies showed that such inferences can be inaccurate and operate implicitly (i.e., without intentional and direct control; Fazio et al., 1995; Greenwald and Banaji, 1995). Inferences about others concerning evaluations with some degree of favor or disfavor (e.g., good/bad, pleasant/unpleasant) that occur outside of conscious awareness and control are defined as implicit attitudes (Greenwald and Banaji, 1995). Implicit attitudes are assessed using indirect measures. Unlike explicit measures (i.e., self-reports) in which the content is assessed directly, implicit measures infer constructs of interest through behavioral performances (e.g., individuals’ response latency or errors in specific tasks). One of the most widely used indirect measures to assess implicit attitudes is the Implicit Association Test (IAT; Greenwald et al., 1998).

Research showed that implicit attitudes can predict variations in behavior across a variety of topics, in many cases above and beyond explicit measures (Fazio et al., 1995; Dovidio et al., 1997; Greenwald and Nosek, 2008; Greenwald et al., 2009). Implicit attitudes have been indeed shown to have a role in predicting discriminatory behaviors, including biased medical decisions (Green et al., 2007), lethal use of force by police (Hehman et al., 2018), and hiring discrimination (Moss-Racusin et al., 2012). Furthermore, they were also found to correlate with physiological markers of empathy (Avenanti et al., 2010).

Over the past decades, several interventions have been proposed in the literature to produce changes in implicit attitudes (Dasgupta and Greenwald, 2001; Blair, 2002; Gawronski and Bodenhausen, 2006; Sritharan and Gawronski, 2010) and mitigate their potential influence on behavior (Guedj et al., 2021a,b; Marini and Banaji, 2022b). This research showed that implicit attitudes can be temporally reduced (Dasgupta and Greenwald, 2001; Marini et al., 2012; Lai et al., 2016) by a variety of factors, ranging from behavioral interventions (e.g., educational programs: Kawakami et al., 2000; Rudman et al., 2001) to the use of neuroscience techniques (e.g., noninvasive brain stimulation: for a review, see Marini et al., 2018). For example, it has been shown that implicit racial attitudes can be temporarily shifted by exposing people to counter-stereotypical exemplars (Marini et al., 2012; Lai et al., 2014), producing changes in their emotional states (DeSteno et al., 2004), or setting equalitarian goals (Legault et al., 2011; Mann and Kawakami, 2012). Similar changes have been observed also using noninvasive brain stimulation techniques (Marini et al., 2018), such as transcranial magnetic stimulation (TMS) and transcranial direct-current stimulation (tDCS). Sellaro et al. (2015), for instance, showed that interfering with the activity of the medial prefrontal cortex (mPFC) *via* tDCS decreased implicit attitudes toward outgroup members.

### Using virtual reality to change implicit attitudes

More recently, studies have shown that implicit attitudes can be shifted also by employing immersive virtual reality (VR). VR represents a powerful tool for embodying people into a “different” body (virtual embodiment) and changing their body representation. When people are virtually embodied with a body different from their own, they tend to exhibit behaviors associated with attributes and characteristics of that body (Banakou et al., 2013): An effect known as Proteus Effect (Yee and Bailenson, 2007).

Studies showed that VR embodiment allows to produce changes in the social processing of individuals and to modulate their implicit attitudes against outgroup members (Peck et al., 2013; Banakou et al., 2016; Salmanowitz, 2018). For example, Peck et al. (2013) demonstrated that the embodiment of White participants in a Black virtual body, for as short as 12 min, produced a reduction of their implicit pro-White attitudes. Similarly, Banakou et al. (2016) conducted a study in which participants performed a series of Tai Chi movements displayed by a virtual teacher. Results showed that not only implicit pro-White attitudes decreased more for participants embodied in a Black than a White virtual body, but also that this effect was still present 1 week after the intervention. In both studies, participants could look at their virtual body both from a first-person perspective, by directly looking down toward themselves, and reflected in a mirror placed in front of them in the virtual environment.

Subsequent research showed that several characteristics of the VR setup seem to have a role in producing a modulation of implicit attitudes (Tassinari et al., 2022b). Indeed, a reduction in implicit attitudes is more likely to occur among those participants who liked their virtual body (Hasler et al., 2017), when the virtual environment had a positive, as opposed to a negative, valence (Banakou et al., 2020), or when the virtual social context was cooperative (Patané et al., 2020). Conversely, other variables, such as the lack of physical similarity between participants’ real body and virtual body (Banakou et al., 2013) or the number of embodiment exposures (Banakou et al., 2016) seems to have no specific influence in reducing implicit attitudes.

### Overview of the study

The main goal of the present study was to further examine conditions under which VR can change implicit racial attitudes. In previous studies, embodiment was achieved by synchronizing the movements of the avatar in the virtual environment with those of the participants who could concurrently see their actions both from the avatar’s perspective and in a virtual mirror placed in the scene (Peck et al., 2013; Banakou et al., 2016, 2020; Hasler et al., 2017; Salmanowitz, 2018). Participants had thus two different visual perspectives on their actions: a first-person perspective given by the synchronization of their movements with those of the virtual avatar and the frontal view of their same movements provided by the virtual mirror. This raises the question as to what is the contribution of the additional view of one’s own movements provided by the virtual mirror and whether this is a crucial factor to elicit changes in implicit attitudes.

To address this question, we used immersive VR to embody White, Caucasian female participants in a Black or White female avatar. Participants wore a body-tracking suit that provided real-time motion capture, so that they could see their virtual body moving synchronously with their real body. To draw attention to their novel virtual body, we required participants to perform a series of manual tasks (Fregna et al., 2022) during the virtual embodiment.

Our study followed a mixed design with two between-subjects factors (condition: “first-person and mirror” vs. “first-person only”; avatar: Black vs. White) and one within-subjects factor (session: pre-intervention vs. post-intervention). That is, each participant was randomly assigned to one of the two experimental conditions and embodied in one of the two avatars. For all participants, implicit racial attitudes were assessed using the Implicit Association Test (IAT) before and immediately after the VR intervention. Participants in the “first-person and mirror” condition could see their virtual body both by looking down toward themselves and reflected in a mirror placed in front of them in the virtual environment, while participants in the “first-person only” condition could instead see their virtual body only from a first-person perspective (i.e., by looking down toward themselves) as no mirror was present in the VR scene.

## Materials and methods

### Participants

A total of 64 volunteers (all female, White, Caucasian; age range 22–38) took part in the present study. Participants were naive to the purpose of the study. The study and all experimental procedures were approved by the local ethics committee (Comitato Etico della Provincia di Ferrara, approval number: 170592). All participants gave their written informed consent and they were paid for their participation.

### Procedure

After signing the informed consent, participants wore a motion capture suite with reflective markers attached on the main joints by means of Velcro straps and completed an Implicit Association Tests (Greenwald et al., 1998) assessing their implicit racial attitudes (race attitude IAT). Then, they went through a VR session during which they were immersed in the virtual environment for approximately 15 min. Finally, they completed again the IAT and filled out a questionnaire evaluating the degree of embodiment experience during the experiment (Gonzalez-Franco and Peck, 2018).

During the VR session, participants comfortably sat in a chair in front of a table. In the VR environment, participants were placed in front of a (virtual) table as well. The experimenter then used calibration routines programmed in our system to set the height and distance of the table in the VR environment to match those of the real table that the participant was facing. In this manner, when touching the table in the VR environment the participant also experienced a sensation of touch produced by the real table. This step was implemented, based on previous results showing that the experience of multi-modal (in our case, vision, touch, and proprioception) matching cues enhance the feelings of embodiment, presence and immersion of subjects in a VR environment (Gallace et al., 2012; Martin et al., 2022). Our immersive virtual environment was developed in C# using the Unity 3D game engine^1^ and the SteamVR plugin (Valve Corporation, Bellevue, United States). It consisted of a cozy home interior with windows showing a beachside scenario (Scandinavian Interior Archviz purchased from the Unity Asset Store) and we displayed it to participants by means of an Oculus Rift (Meta, Menlo Park, CA) Head Mounted Display (HMD).

While immersed in the virtual environment, the subjects performed three different tasks (Fregna et al., 2022), with the purpose of acting with and paying attention to their virtual body. Task 1 (Glasses): The task started with four pedestals presented on the table. The pedestals were distributed along a circle centered on the participants’ body at equal angular displacements. A glass then appeared on one randomly selected pedestal and the participants had to push it down using the hand closer to the pedestal on which it appeared. Task 2 (Cloud): At the beginning of trial a cloud of small bubbles appeared, which pop upon touching. The cloud was placed either to the right or to the left of the participants and they had to pop all of the bubbles with the corresponding hand. Task 3 (Ball in hole): For this task, a box-like support with a pocket at its center was placed on the virtual table. At the beginning of each trial, a tennis ball was placed on this support either to the right or left of the participants and they had to gently push the ball into the hole with their corresponding hand.

**Figure 1:**
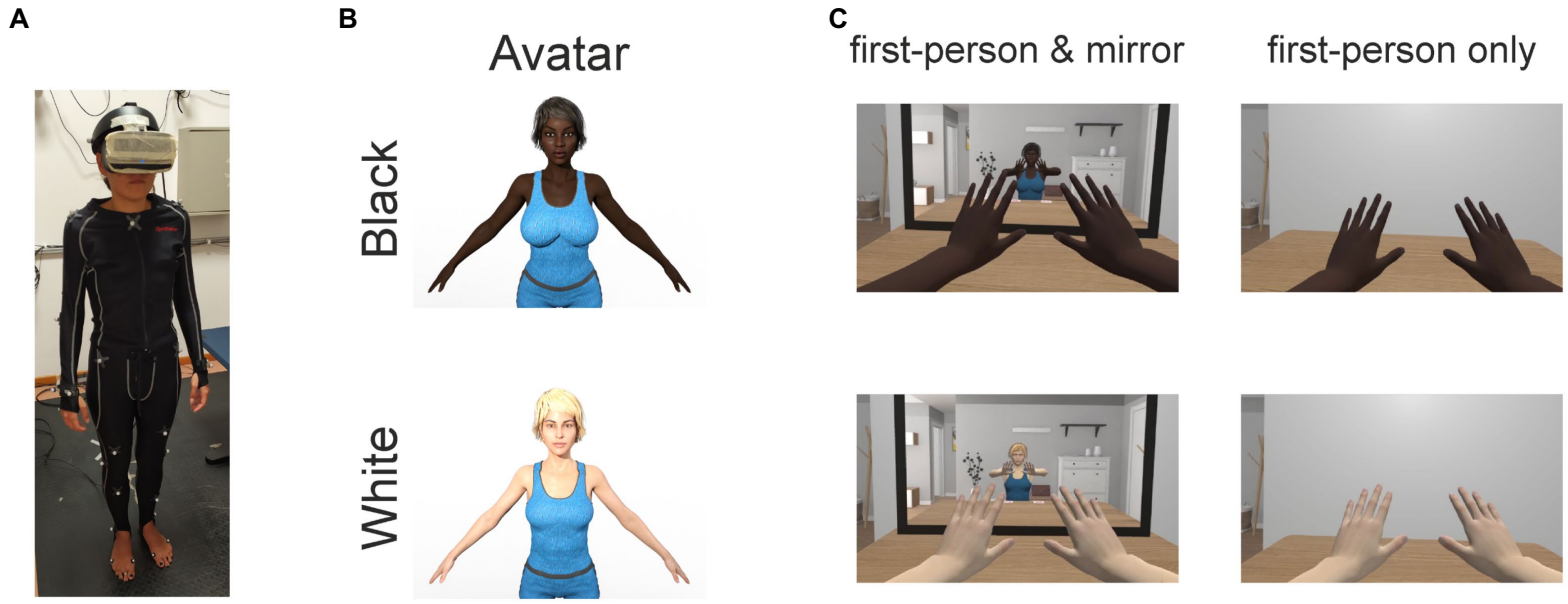
Experimental setup and conditions. **(A)** Motion capture suite with reflective markers and head-mounted display wore by participants in our experiments. **(B)** Photorealistic Black (upper row) and White (bottom row) avatars used in our experiments. **(C)** Experimental conditions. Participants in the “first-person and mirror” condition (left column) could see their virtual body both from a first-person perspective (i.e., by looking down toward themselves) and reflected in a mirror placed in front of them in the VR environment. Participants in the “first-person only” condition (right column) could see their virtual body only from a first-person perspective (i.e., by looking down toward themselves) as no mirror was placed in the VR environment (right column).

During task performance the participants wore a motion capture suit (Figure 1A). Their movements were measured by means of an OptiTrack motion capture system (NaturalPoint, Inc., Corvallis, OR, United States) equipped with 15 Flex 13 cameras and they were used to animate in real-time a photorealistic avatar (Figure 1B). The virtual camera of the HMD was placed in the head of the avatar as to give the impression of effectively being in the avatar’s body. Each participant performed one of two primary conditions (“first-person and mirror” or “first-person only”) and was embodied in one of two avatars (Black or White). Participants in the “first-person and mirror” condition, could see their virtual body both by looking down toward themselves and reflected in a mirror placed in front of them in the virtual scene (left column in Figure 1C). Participants in the “first-person only” condition, could see their body only by looking down toward themselves as no virtual mirror was placed in the VR environment (right column in Figure 1C). For the White avatar, participants were embodied in a white Caucasian avatar (Monique8 HD from the DAZ asset store, Salt Lake City, UT, United States), while participants for the Black avatar were embodied in an African American avatar (Imani8 HD also from the DAZ store).

### Measures

#### Implicit association test

The Implicit Association Test (IAT; Greenwald et al., 1998) is a widely-used and validated implicit measure that assesses psychological construct (e.g., attitudes, stereotypes) indirectly by measuring how quickly and accurately a person can categorize and associate stimuli related to two conceptual categories and two attributes. In this study, participants completed a race attitude IAT assessing their implicit racial attitudes. Specifically, the race attitude IAT evaluated the automatic strength of association between the categories Black People and White People and the evaluative attributes good and bad.

The IAT followed the standard procedure described in Nosek et al. (2005). Participants categorized stimuli representing two categories and two attributes in two different sorting conditions by pressing one of two keys. In one condition, participants categorized pictures of Black individuals and positively-valenced words (e.g., joy and love) with one response key, while categorizing pictures of white individuals and negatively-valenced words (e.g., agony and hate) by using another response key. In the other condition, they categorized the same stimuli but with a different response key configuration: this time pictures of Black individuals and negatively-valenced words were categorized with one key, whereas pictures of White individuals and positively-valenced words with the other. The order of these two conditions was randomized across participants. Stimuli were presented one at a time at the center of a computer screen.

We computed IAT scores according to the algorithm described by Greenwald et al. (2003). That is, we divided the difference in mean response between the two IAT sorting conditions by the participant’s latency standard deviation inclusive of the two conditions. Responses faster than 350 ms and slower than 10,000 ms were removed, and errors were replaced with the mean of the correct responses in that response block plus a 600 millisecond penalty. IAT score could range from +2 to-2, with zero indicating no implicit preference between Black and White people. Positive scores indicated implicit pro-White attitudes, while negative scores indicated implicit pro-Black attitudes. Participants with IAT scores or differences in scores deviating more than 2SD from their group’s mean were considered outliers and were not considered further. A total of 58 participants were included in our analysis.

#### Embodiment questionnaire

To evaluate the degree of embodiment during the experiment we used a subset of a standardized questionnaire proposed by Gonzalez-Franco and Peck (2018). The questionnaire was administered in Italian at the end of the session and it consisted of 11 questions (see section 1 in the Supplementary Material) corresponding to questions Q1, Q2, Q3, Q6, Q7, Q8, Q9, Q17, Q18, Q19, and Q20 in Gonzalez-Franco and Peck (2018). The subjects could respond to each question by checking one out of 7 possible choices corresponding to a 7 point Likert scale ranging from-3 to 3, with-3 indicating strong disagreement and 3 indicating strong agreement with the statement. Ratings were used to quantitatively evaluate three aspects of the embodiment experience, namely Ownership, Agency, and Appearance, that, following Gonzalez-Franco and Peck (2018), were computed in the following manner:

- Ownership: (Q1 - Q2) - Q3
- Agency: Q6 + Q7 + Q8 – Q9
- Appearance: Q17 + Q18 + Q19 + Q20

## Results

### Implicit association test

Overall, participants showed positive IAT scores before the VR intervention, indicating strong implicit pro-White attitudes (*M* = 0.61, SD = 0.33, Cohen’s *d* = 1.85, *t*(57) = 14.165, *p* < 0.001). Mean IAT scores and their standard deviation for each condition before and after VR intervention are reported in Supplementary Table S1.

Following the analysis procedure applied in previous VR studies investigating implicit attitudes (Peck et al., 2013; Banakou et al., 2016), IAT scores after the VR intervention (post-intervention) were submitted to an ANCOVA with avatar (Black vs. White) and condition (first-person and mirror vs. first-person only) as fixed factors. Scores before the VR intervention (pre-intervention) were included in the analysis as a covariate. Results revealed a significant interaction between factors avatar and condition [*F*(1,53) = 4.624, *p* < 0.05, ηp2 = 0.080], indicating that implicit racial attitudes after VR intervention were modulated by these two factors. No significant main effect of the factors avatar [*F*(1,53) = 0.949, p = n.s., ηp2 = 0.018] or condition [*F*(1,53) = 0.025, p = n.s., ηp2 = 0.000] was observed. The covariate showed a significant effect [*F*(1,53) = 11.236, p < 0.001, ηp2 = 0.175], indicating a relevant contribution in the model.

**Figure 2:**
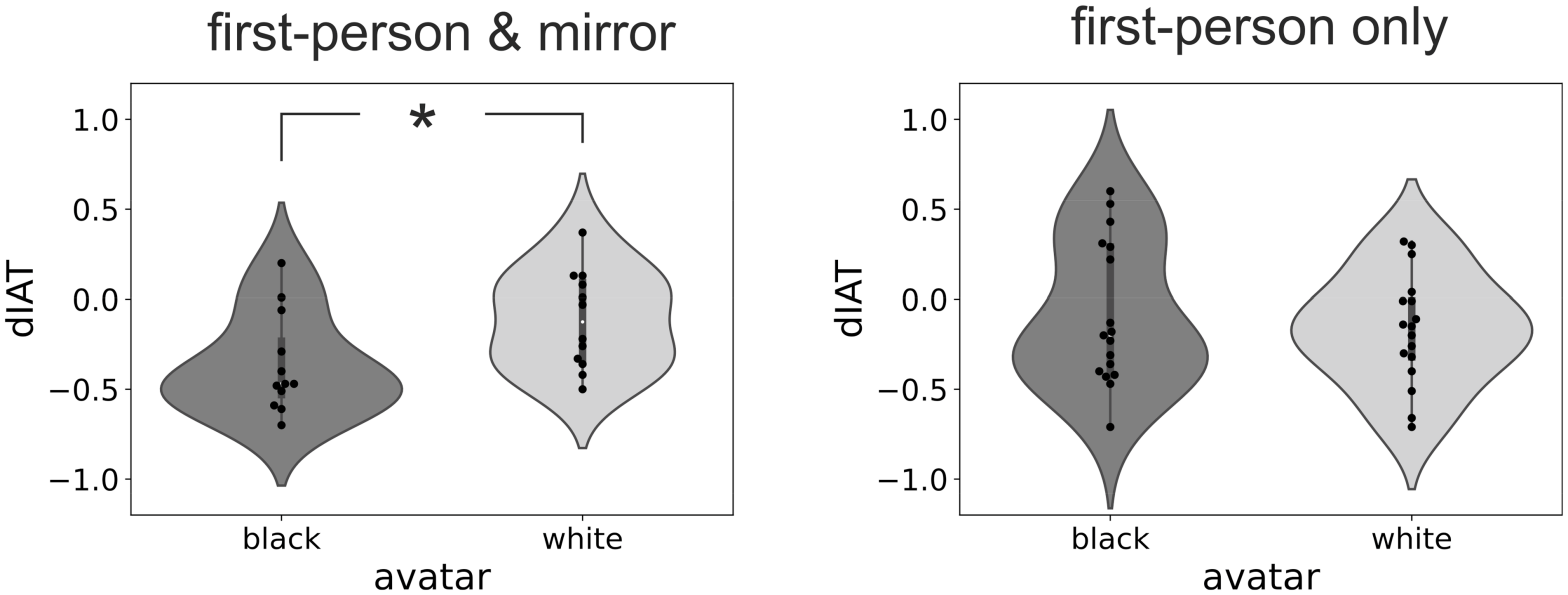
Changes in IAT scores – In all panels the black dots represent the individual differences in IAT scores (dIAT) after and before virtual embodiment for the Black (dark gray bar) and White (light gray bar) avatar. The left panel shows results for participants that could see their virtual bodies both from a first-person perspective and in a mirror, while the right panel shows results for participants that could see their virtual body only from a first-person perspective. The thick vertical bars represent the first and third quantiles and the white dots the median of the distribution. Significant differences are highlighted by an asterisk (*).

To study the interaction between the factors avatar and condition, we then compared the difference in IAT scores, measured after and before VR embodiment, in the Black and White avatar for each condition. Results showed that, in the “first-person and mirror” condition, the embodiment in a Black avatar produced a stronger reduction of implicit pro-White attitudes than when they were embodied in a White avatar (left panel in Figure 2; *t*(22) = 2.22, *p* < 0.05). No difference between avatars was instead observed in the “first-person only” condition (right panel in Figure 2; *t*(32) = −0.68, *p* = n.s.). These results show a reduction of implicit pro-White attitudes when participants are embodied in a Black avatar (compared to a White avatar) but only if they can see their virtual body both from a first-person perspective and in a mirror.

### Embodiment questionnaires

**Figure 3:**
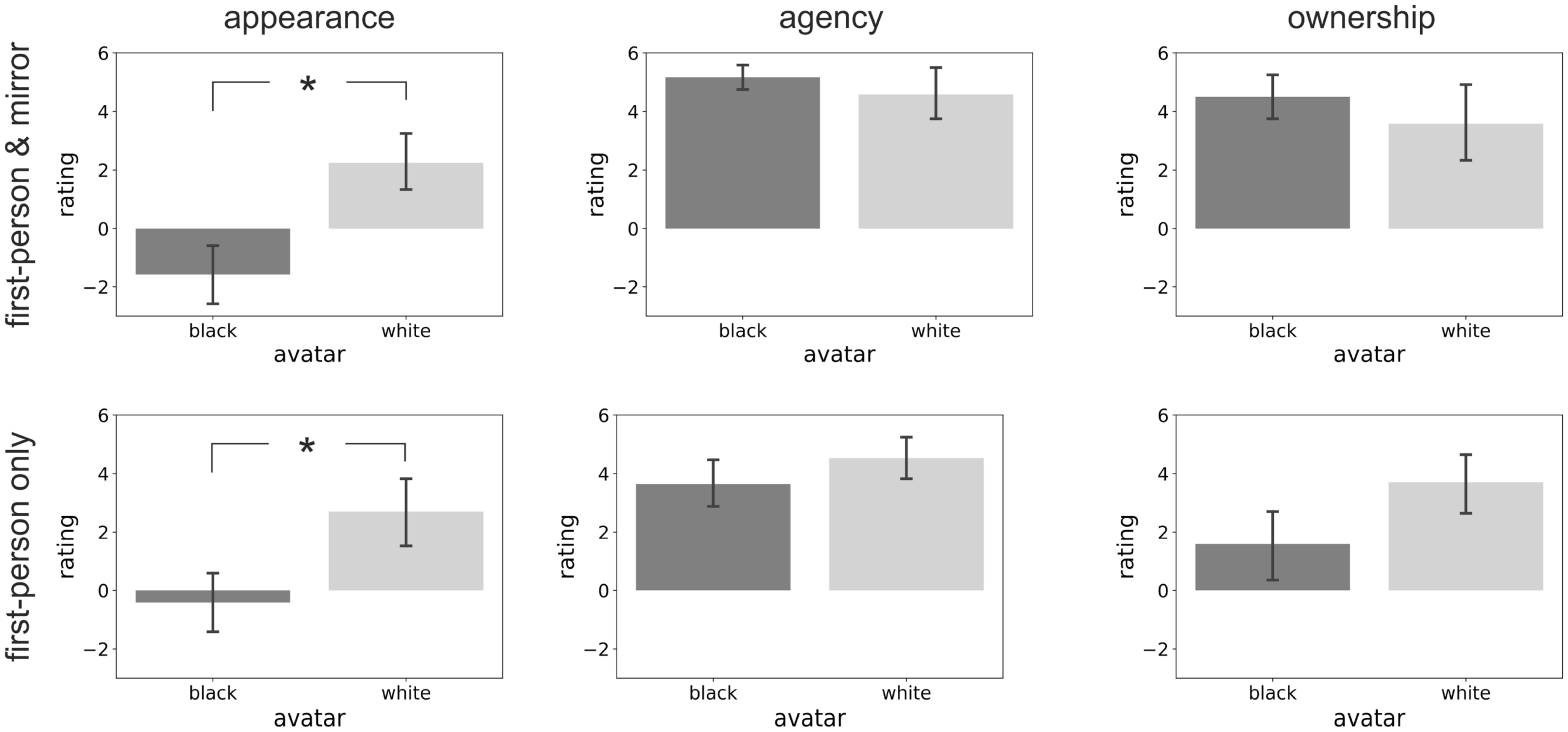
Results of the appearance, agency and ownership questionnaires – The six panels show the appearance (left column), agency (middle column) and ownership (right column) ratings obtained in the post-experiment questionnaires when participants could see their body in both the first-person perspective and in a mirror (“first-person and mirror” condition, upper row) and when they could see it only from a first-person perspective (“firstperson only” condition, lower row). Vertical bars represent standard error. Significant differences are highlighted by an asterisk (*).

A t-test analysis of the “appearance” scores revealed that they were higher for the White than for the Black avatar (Left column in Figure 3) both in the “first-person and mirror” (Black: −1.58 ± 3.63, White: 2.25 ± 3.55; *t*(22) = −2.62, *p* < 0.05) and in the “first-person only” (Black: −0.41 ± 4.05, White: 2.71 ± 4.77; *t*(32) = −2.06, *p* < 0.05) conditions. The same analysis on the “agency” scores revealed instead no difference between the Black and White avatars neither in the “first-person and mirror” (Black: 5.17 ± 1.53, White: 4.58 ± 3.34; *t*(22) = 0.55, *p* = n.s.) nor in the “first-person only” (Black: 3.65 ± 3.32, White: 4.53 ± 3.14; *t*(32) = −0.8, *p* = n.s.) condition. Similar results were obtained for the “ownership” scores (first-person and mirror: Black: 4.5 ± 2.58, White: 3.58 ± 4.54; *t*(22) = 0.61, p = n.s.; first-person only: Black: 1.59 ± 4.72, White: 3.71 ± 3.98; *t*(32) = −1.41, *p* = n.s.). Taken together, these results indicate that participants were aware of the different bodily characteristics of the avatars as the appearance ratings were significantly different between Black and White avatars in both the “first-person and mirror” and “first-person only” conditions. However, the reduction of implicit pro-White attitudes after the VR intervention with a Black avatar cannot be due to a potential different experience in acting in the virtual environment as the agency and ownership ratings were not significantly different between the Black and White avatars in neither condition.

## Discussion

The main purpose of the present study was to examine the conditions under which VR embodiment in an outgroup member can change implicit racial attitudes. To this aim, we conducted an experiment in which participants completed an IAT assessing racial attitudes both before and after a VR intervention. In the VR intervention, we manipulated participants’ perspective of their virtual body: in one condition (“first-person and mirror”), they could see their virtual body both from a first-person perspective (i.e., both by looking down toward themselves) and reflected in a mirror placed in front of them in the virtual environment, while in another condition (“first-person only”), they saw their virtual body only from a first-person perspective (i.e., by looking down toward themselves) as no mirror was placed in the VR environment. Results showed that the implicit pro-White attitudes of participants embodied in a Black avatar decreased in a significant manner, compared to a White avatar, when they could see their virtual body both from a first-person perspective and in a mirror. We observed no such significant decrease when participants saw their virtual body only from a first-person perspective. These results indicate that participants’ perspective of their virtual body in the VR environment might be a critical factor to change implicit racial attitudes. Specifically, they suggest that seeing the virtual body in a mirror might be a crucial factor to produce such change.

One potential explanation for these results is that the presence of the virtual mirror in the “first-person and mirror condition” gave participants a visual feedback not only of their bodily movements and features (e.g., their skin tone) but also of their facial features, increasing thus the perception to be embodied in a virtual body of a different race. Previous studies showed that facial characteristics compared to skin color play a major role in the perception and recognition of Black individuals (Dixon and Maddox, 2005; Ronquillo et al., 2007; Bar-Haim et al., 2009; Brooks and Gwinn, 2010; Strom et al., 2012). For example, in a study in which participants were asked to rate the racial prototypicality of individuals belonging to different race groups, White participants perceived Black people as more or less racially prototypical relying more on their facial features rather than on their skin color (Strom et al., 2012). Similarly, in a study in which the skin color of Black individuals was manipulated, results showed that facial features played a bigger role, compared to skin tone, in the recognition of Black individuals (Bar-Haim et al., 2009). It is thus conceivable that, in our experiments, seeing the virtual face reflected in a mirror placed in the VR environment may have induced in the participants a stronger recognition of their virtual body as an outgroup member, producing a change in their implicit pro-White attitudes. Notably, it has been demonstrated that facial features are critical also to activate evaluative judgments (for reviews, see Maddox, 2004; Kleider-Offutt et al., 2017). For example, it has been shown that Black people with specific facial characteristics (e.g., a wide nose and full lips) were readily recognized and categorized as Black individuals, but they were also more likely to be associated with stereotypical attributions (Blair et al., 2004; Kleider et al., 2012; Knuycky et al., 2014). These findings suggest that the lack of a reduction in implicit racial attitudes after the VR intervention in which participants could see their virtual body only from a first-person perspective found in our study, may be due to the fact that participants could not see their virtual face and thus activate the evaluative attributions associated to the outgroup.

An alternative, non-mutually exclusive, explanation is that the presence of the virtual mirror in the “first-person and mirror” condition recruited, compared with the “first-person only” condition, additional representations of one’s own body that enhanced the efficacy of the VR intervention. Indeed, results in somatoparaphrenia patients suggest that first-person and mirror-view of one’s own actions rely on partially different neuronal substrates. Somatoparaphrenia is a condition, predominantly associated with brain damages to the right hemisphere, in which patients experience disownership of left-sided body parts (for review see, Vallar and Ronchi, 2009). That is, the delusional feeling that they belong to someone else. Notably, the feeling of disownership significantly decreased when somatoparaphrenia patients observed their body in a mirror (Fotopoulou et al., 2011; Jenkinson et al., 2013). Interestingly, also the opposite dissociation (i.e., disownership of one’s own body when viewed in a mirror but not in direct, first-person, view) has been reported (Breen et al., 2000). A complementary role of the first-person and mirror views of one’s own bodily movements is also suggested by behavioral experiments showing a greater visual sensitivity to either the former (Campanella et al., 2011) or the latter (Prasad and Shiffrar, 2009) stimulus presentation, depending on the task. Interestingly, the existence of partially dissociated neuronal and behavioral representations of the egocentric and allocentric points of view has been shown also for observation of others’ actions (Maeda et al., 2002; Shmuelof and Zohary, 2008; Caggiano et al., 2011, 2015; Casile, 2013, 2022). Taken together, the experimental results reviewed above suggest that the first-person and mirror views of one’s own actions have partially non-overlapping neuronal and cognitive representations. It is thus conceivable that, when both are present, as in the “first-person and mirror” condition in our experiments, the brain has access to additional information that enhance the experience of being embodied in a virtual avatar. This enhancement is not consciously perceived, as participants’ explicit ownership and agency scores are not different between the “first-person and mirror” and “first-person only” conditions but it nonetheless influences their implicit, unconscious racial attitudes.

In addition to immersive reality, a widely-used paradigm to elicit the embodiment of fake bodily parts is the rubber hand illusion (RHI: Botvinick and Cohen, 1998). In the RHI, subjects see a lifelike rubber hand placed in front of them being stroke with a brush simultaneously with their corresponding unseen hand. After few minutes of simultaneous stimulation of the rubber and their unseen hand, the subjects report a strong sense of embodiment of the rubber hand and report it as being their own (Botvinick and Cohen, 1998). In the standard RHI paradigm, the to-be-embodied, rubber hand is seen from the same subjective perspective as in the “first-person only” condition in our experiments. The question thus arises as to why we found no significant modulation of the implicit bias in that condition, while a modulation of implicit associations has been consistently reported in RHI studies (Farmer et al., 2012, 2014; Maister et al., 2013). An important difference between our “first-person only” condition and the RHI paradigm is that, participants in the latter, experience, in addition to the first-person representation of the rubber hand, also a consistent tactile stimulation (i.e., the simultaneous stroking of both the real and rubber hands). The visual representation, *per se*, is not sufficient to create an embodiment of the rubber hand. In our “first-person only” paradigm, while participants experience a consistent proprioceptive feedback (i.e., they see their virtual body move when they move their real body), they cannot necessarily experience a tactile feedback from their interactions with objects in the virtual environment. The addition of the mirror in the “first-person and mirror” condition might have thus contributed to, at least partially, overcome this “sensory conflict” by activating complementary/additional one’s own body’s representations. In keeping with the idea that a mirror can indeed activate additional internal bodily representations, is the experimental finding that while the RHI is virtually abolished when the rubber is rotated 180 degree from the subjects’ body and it is thus seen from a frontal rather than a first-person perspective (Costantini and Haggard, 2007), it is instead fully established when the rubber hand or a whole-body mannequin are presented from the same frontal perspective by means of a mirror (Bertamini et al., 2011; Jenkinson et al., 2013; Preston et al., 2015).

Our study has also limitations that are worth discussing. First, it provides no understanding of the mechanisms mediating the modulation of implicit bias that we observed. To this end, a potentially promising direction for future studies, might be the exploration of moderating factors in prejudice reduction in VR (Tassinari et al., 2022b). Presently available evidence seems to suggest that the emotional valence of the VR experience has a role in this process (e.g., Kalyanaraman et al., 2010; Banakou et al., 2020) and further studies are needed to fully elucidate this point. Second, the scope of our questionnaires was limited to measure participants’ subjective ratings of ownership, agency and appearance during the VR embodiment. There are indications that, at the individual level, personality traits (e.g., empathy or nervousness during the VR immersion) might contribute to enhance prejudice reduction in VR (Peck et al., 2013; Chen et al., 2021) and future studies might want to include questionnaires to evaluate participants also along those dimensions. Finally, future studies should explore whether the changes in the race IAT observed here using VR are also associated with changes in behavior. Recent research indeed showed that changes in implicit measures as the IAT are possible, but those changes might not readily translate into changes in behavior (Forscher et al., 2019).

In summary, our results have important implications for the creation of VR interventions aimed at changing implicit racial attitudes as they provide relevant information about conditions under which these changes can occur. In particular, they suggest that the possibility of viewing one’s own body and actions in a virtual environment both from a first-person perspective and reflected in a mirror might be important to produce chances in implicit attitudes. Results presented here represent a further step forward for understanding the malleability of implicit racial preferences using VR and for developing future basic research to clarify the mechanisms of such change. Future studies comparing different VR interventions are needed to further determine the most effective practices in using VR to change implicit attitudes. For example, VR could be also used to create virtual interactions with outgroup members (Breves, 2020; Tassinari et al., 2022a) or it may be used together with behavioral interventions aimed at reducing implicit pro-White attitudes (Lai et al., 2014) to enhance their effects by providing a more vivid and realistic experience.

## Data availability statement

The raw data supporting the conclusions of this article will be made available by the authors, without undue reservation.

## Ethics statement

The studies involving human participants were reviewed and approved by Comitato Etico della Provincia di Ferrara, approval number: 170592. The patients/participants provided their written informed consent to participate in this study.

## Author contributions

MM and AC conceived and designed the study, implemented the study, analyzed the data, interpreted the data, drafted the manuscript, and revised and approved the manuscript. All authors contributed to the article and approved the submitted version.

## Funding

This work was partially supported by a grant from the Bial Foundation (grant number 344/18).

## Conflict of interest

The authors declare that the research was conducted in the absence of any commercial or financial relationships that could be construed as a potential conflict of interest.

## Publisher’s note

All claims expressed in this article are solely those of the authors and do not necessarily represent those of their affiliated organizations, or those of the publisher, the editors and the reviewers. Any product that may be evaluated in this article, or claim that may be made by its manufacturer, is not guaranteed or endorsed by the publisher.

## Footnotes

^1^ www.unity3d.com
