# Supplementary material for: I can see my virtual body in a mirror: The role of visual perspective in changing implicit racial attitudes using virtual reality
Source: Front Psychol. 2022 Nov 28;13:989582. doi: 10.3389/fpsyg.2022.989582 (PMC9742480; doi:10.3389/fpsyg.2022.989582)
Supplement: Supplementary file 1 [file Data_Sheet_1.PDF]

Supplementary Material

1 Embodiment Questionnaire

The embodiment questionnaire was administered in Italian. It consisted of 6 questions derived from questions Q1, Q2, Q3, Q6, Q7, Q8 Q9, Q17, Q18, Q19 and Q20 in (Gonzalez-Franco & Peck, 2018).

“Durante l’esperimento ci sono stati momenti in cui ...

Q1. Ho avuto la sensazione che il corpo virtuale fosse il mio corpo”

Q2. Ho avuto la sensazione che il corpo virtuale fosse di qualche altro/a”

Q3. Ho avuto la sensazione di avere più di un corpo”

Q4. Ho avuto la sensazione di controllare il corpo virtuale come se fosse il mio corpo”

Q5. I movimenti del corpo virtuale erano controllati da quelli del mio corpo”

Q6. Ho avuto la sensazione che i movimenti del corpo virtuale influenzassero i miei movimenti”.

Q7. Ho avuto la sensazione che il corpo virtuale si muovesse da solo”.

Q8. Ho avuto la sensazione che il mio corpo si trasformasse nel corpo di un avatar”.

Q9. Ad un certo punto ho avuto la sensazione che il mio corpo reale iniziasse ad assumere la postura del corpo virtuale che vedevo”.

Q10. Ad un certo punto ho avuto la sensazione che il mio corpo virtuale assomigliasse al mio corpo in termini di forma, carnagione ed altre caratteristiche visive”.

Q11. Ho avuto la sensazione di indossare vestiti diversi da quelli che avevo quando sono arrivato in laboratorio”.

English Version:

“During the experiment there were moments in which…

Q1. I felt as if the virtual body was my body”

Q2 I felt as if the virtual body was someone else’s”

Q3. I felt as if I had more than one body”

Q4. I felt like I could control my virtual body as if it was my own body"

Q5. I felt as if the movements of my virtual body were caused by my movements"

Q6. I felt as if the movements of the virtual body were influencing my movements"

Q7. I felt as if the virtual body was moving by itself"

Q8 – I felt as if my body was turning into an avatar's body"

Q9 – at some point I felt as if my real body was starting to take on the posture of the virtual body"

Q10 – at some point I felt as if my virtual body resembled my own body, in terms of shape, skin tone, or other visual features"

Q11 – I felt like I was wearing different clothes from when I came to the laboratory"

2 Mean IAT scores before intervention

Table S1 – This table shows the mean IAT scores and their standard deviations for each of the four experimental groups before and after the VR intervention.

	**First-person & mirror**			**First-person only**	
	**black**	**white**		**black**	**white**
**Pre-intervention**	0.76±0.20	0.69±0.2		0.53±0.42	0.54±0.32
**Post-intervention**	0.39±0.24	0.58±0.28		0.44±0.13	0.37±0.35

References

Gonzalez-Franco, M., & Peck, T. C. (2018). Avatar embodiment. Towards a standardized questionnaire. *Frontiers in Robotics and AI, 5*(74), 1–9. https://doi.org/10.3389/frobt.2018.00074
